# Oxidative stress and its related epigenetic modifications in vascular calcification: mechanisms and advances

**DOI:** 10.3389/fcvm.2025.1662989

**Published:** 2025-10-20

**Authors:** Yanxia Lin, Huanrui Zhang, Yuqi Jiang, Wen Tian

**Affiliations:** Department of Geriatric Cardiology, The First Hospital of China Medical University, Shenyang, Liaoning, China

**Keywords:** oxidative stress, epigenetic modification, vascular calcification, DNA methylation, histone modification, microRNA

## Abstract

Vascular calcification (VC) refers to the pathological deposition of hydroxyapatite within the arterial wall and is characterized by the transdifferentiation of vascular smooth muscle cells (VSMCs) into osteogenic phenotypes. Emerging evidence indicates that oxidative stress plays a pivotal role in the initiation and progression of vascular calcification. Excessive production of reactive oxygen species (ROS) not only activates the expression of calcification-related genes but also promotes VSMC phenotypic switching through diverse epigenetic mechanisms. In this review, we summarize current advances in understanding the interplay between oxidative stress and epigenetic regulation in VC, to provide novel theoretical perspectives on the pathogenesis of this complex vascular disorder.

## Graphical Abstract

**Figure:**
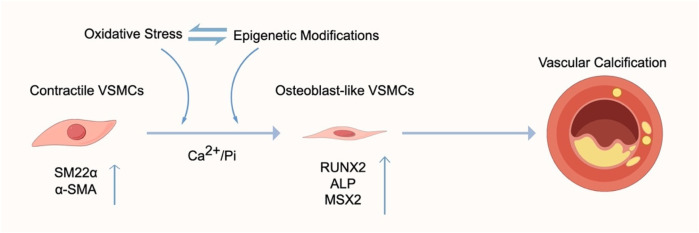

## 1 Introduction

Vascular calcification (VC) is defined as the pathological deposition of hydroxyapatite crystals within the arterial wall. It is recognized as a hallmark of advanced vascular disease and a strong predictor of adverse cardiovascular outcomes (1–3). VC contributes to increased arterial stiffness, systolic blood pressure, and pulse wave velocity (4), thereby exacerbating the morbidity and mortality of cardiovascular diseases (5, 6). Mechanistically, VC mirrors physiological bone formation, with the phenotypic switch of vascular smooth muscle cells (VSMCs) from a contractile to an osteoblast-like phenotype serving as the central process (7). This transition is characterized by the downregulation of contractile marker genes and the upregulation of osteogenic transcription factors, including runt-related transcription factor 2 (RUNX2), Msh homeobox 2 (MSX2), and alkaline phosphatase (ALP), among others (8, 9). Beyond these phenotypic changes, dysregulated biological processes linked to oxidative stress—such as VSMC apoptosis, impaired autophagy, and endoplasmic reticulum stress—also play critical roles in the pathogenesis of VC (10).

Recent evidence highlights the significance of oxidative stress and epigenetic changes in VC development (11, 12). Although the direct interactions between these two processes remain insufficiently explored, their synergistic effects on VSMC function and vascular homeostasis are increasingly recognized. This review aims to clarify how oxidative stress and its related epigenetic changes contribute to VC, offering a comprehensive understanding of this complex condition.

## 2 Oxidative stress and vascular calcification

**Figure 1:**
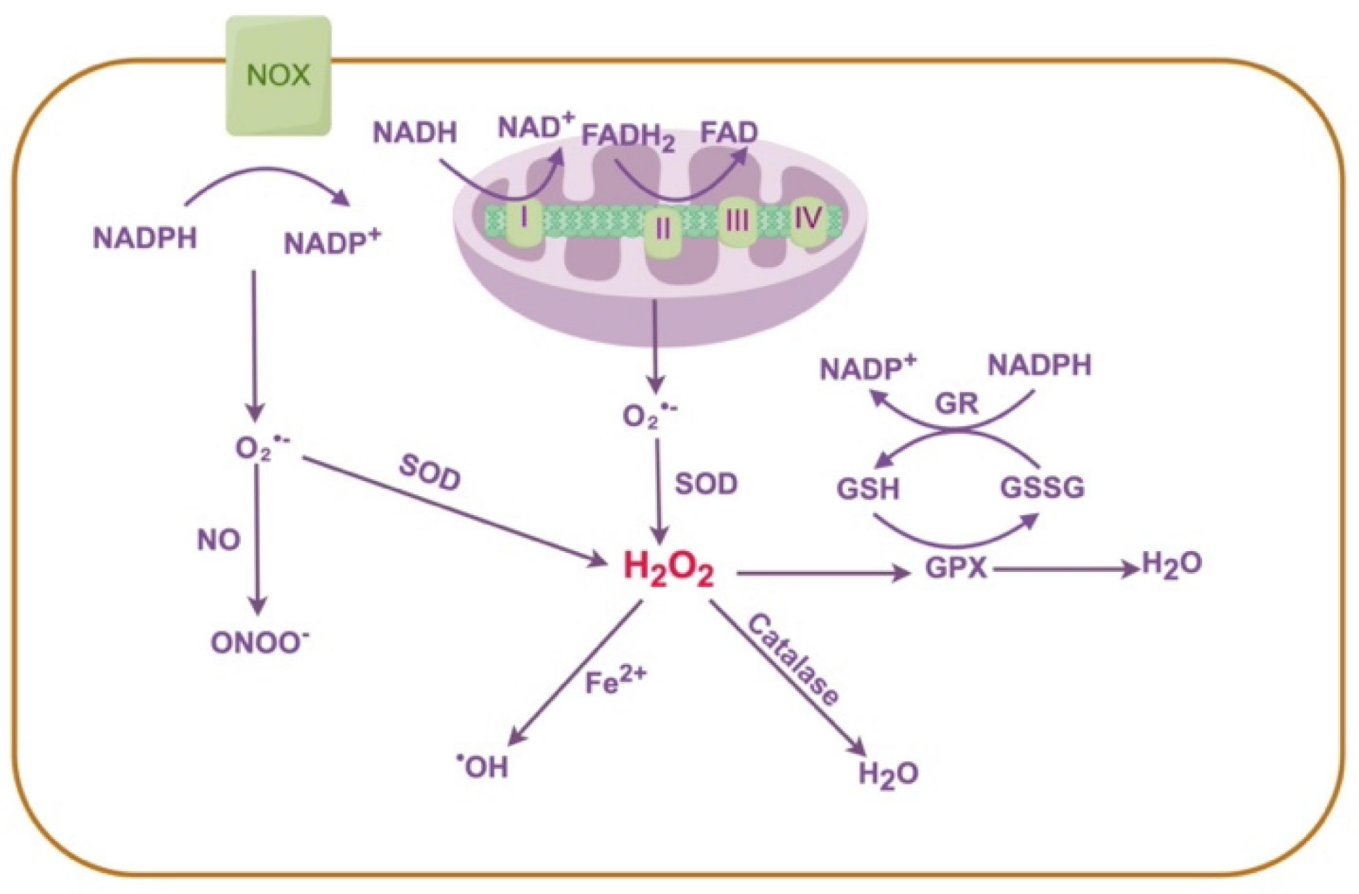
Generation and clearance of ROS. Mitochondrial ETC complexes I and III and NADPH oxidases are major sources of O_2_^•−^. NOX catalyze the oxidation of NADPH to NADP+, generating O_2_^•−^. NO reacts with O_2_^•−^ to form ONOO−. O_2_^•−^ is rapidly converted to H_2_O_2_ by SOD. H_2_O_2_ is further decomposed into H_2_O and O_2_ by catalase, or reduced to H_2_O by GPX using GSH as a substrate, producing GSSG. Meanwhile, GR reduces oxidized GSSG to GSH using NADPH as an electron donor. In the presence of Fe^2+^, H_2_O_2_ undergoes the Fenton reaction to form ^•^OH, which exerts strong oxidative damage. ETC, electron transport chain; NADPH, nicotinamide adenine dinucleotide phosphate; NOX, NADPH oxidases; O_2_^•−^, superoxide anion; NO, nitric oxide; ONOO^−^, peroxynitrite; H_2_O_2_, hydrogen peroxide; ^•^OH, hydroxyl radical; SOD, superoxide dismutase; GSH, glutathione (reduced form); GSSG, glutathione disulfide (oxidized form); GR, glutathione reductase.

Oxidative stress occurs when excessive reactive oxygen species (ROS) accumulate and overwhelm the body's natural antioxidant defenses, resulting in damage to DNA, proteins, and lipids (13). ROS can be broadly classified into free radicals—such as the superoxide anion (O_2_^•−^) and hydroxyl radical (^•^OH)—and non-radical oxidants, including hydrogen peroxide (H_2_O_2_) and peroxynitrite (ONOO−) (14, 15). These reactive molecules are usually neutralized by antioxidant defense systems such as superoxide dismutase (SOD), catalase, and glutathione peroxidase (GPx) (14, 16). Two main sources of ROS in vascular cells are nicotinamide adenine dinucleotide phosphate (NADPH) oxidases (NOX) and mitochondria (17, 18). Mitochondria are the primary sources of cellular ROS (mtROS), generated as byproducts of electron transport chain (ETC) activity (15, 19, 20) (Figure 1). Under physiological conditions, redox balance is maintained through dynamic regulation between ROS production and antioxidant defenses. However, mitochondrial dysfunction leads to excessive ROS accumulation, thereby aggravating oxidative stress (21). Similarly, increased NOX activity and impaired ROS clearance synergistically contribute to vascular oxidative damage and calcification (18, 22). Accumulating evidence highlights oxidative stress as a key driver of VC. ROS overproduction not only promotes VSMC transdifferentiation into osteogenic-like phenotypes but also accelerates the progression of calcification (23, 24). Conversely, interventions that suppress oxidative stress, including antioxidants and ROS inhibitors, have been shown to attenuate VC development (25).

Mitochondria undergo dynamic fission and fusion to preserve their functional integrity. Excessive fission causes fragmentation, reduced bioenergetics, and increased ROS production (26). Dynamin-related protein 1 (DRP1), a key mediator of fission, promotes mitochondrial fragmentation, membrane depolarization, and oxidative stress when overexpressed (27). DRP1 has been implicated in the osteogenic phenotypic switch of VSMCs, and its enrichment at calcified vascular sites has been confirmed. Pharmacological or genetic inhibition of DRP1 attenuates oxidative stress-induced VSMC calcification (28, 29). Notably, quercetin, an antioxidant flavonoid, reduces DRP1 expression and prevents phosphate (Pi)-induced calcification in renal failure rat models, further linking mitochondrial dynamics and oxidative stress to VC (28).

Mitochondrial DNA (mtDNA) is highly vulnerable to oxidative damage because of its proximity to sources of ROS and the absence of protective histones and introns (30). Alterations in mtDNA copy number are considered sensitive biomarkers of oxidative stress (31). Accumulation of oxidative mtDNA damage has been observed in VC and other vascular pathologies (1). DNA polymerase γ (PolG), the only mitochondrial DNA polymerase, is crucial for mtDNA replication, proofreading, and repair. Its exonuclease activity maintains genomic accuracy and prevents mutations. Recent studies show that PolG, along with p53, helps preserve mitochondrial function, reduces oxidative stress, and alleviates VC. Conversely, the loss of this repair ability, as seen in the PolG D257A mutation, accelerates oxidative damage and vascular calcification (32).

The mitochondrial permeability transition pore (MPTP) also plays a crucial role in mitochondrial homeostasis. Elevated Ca^2+^ and oxidative stress promote MPTP opening (33). Transient openings enable solute exchange, while prolonged openings trigger ROS bursts, mitochondrial swelling, Ca^2+^ release, and cell death (34). Inorganic polyphosphate-induced VC is primarily mediated by mitochondrial dysfunction, ATP depletion, and sustained MPTP opening (24, 35, 36). Concurrent accumulation of Ca^2+^ and Pi in the cytoplasm and mitochondria aggravates oxidative stress and drives VC progression (10).

**Figure 2:**
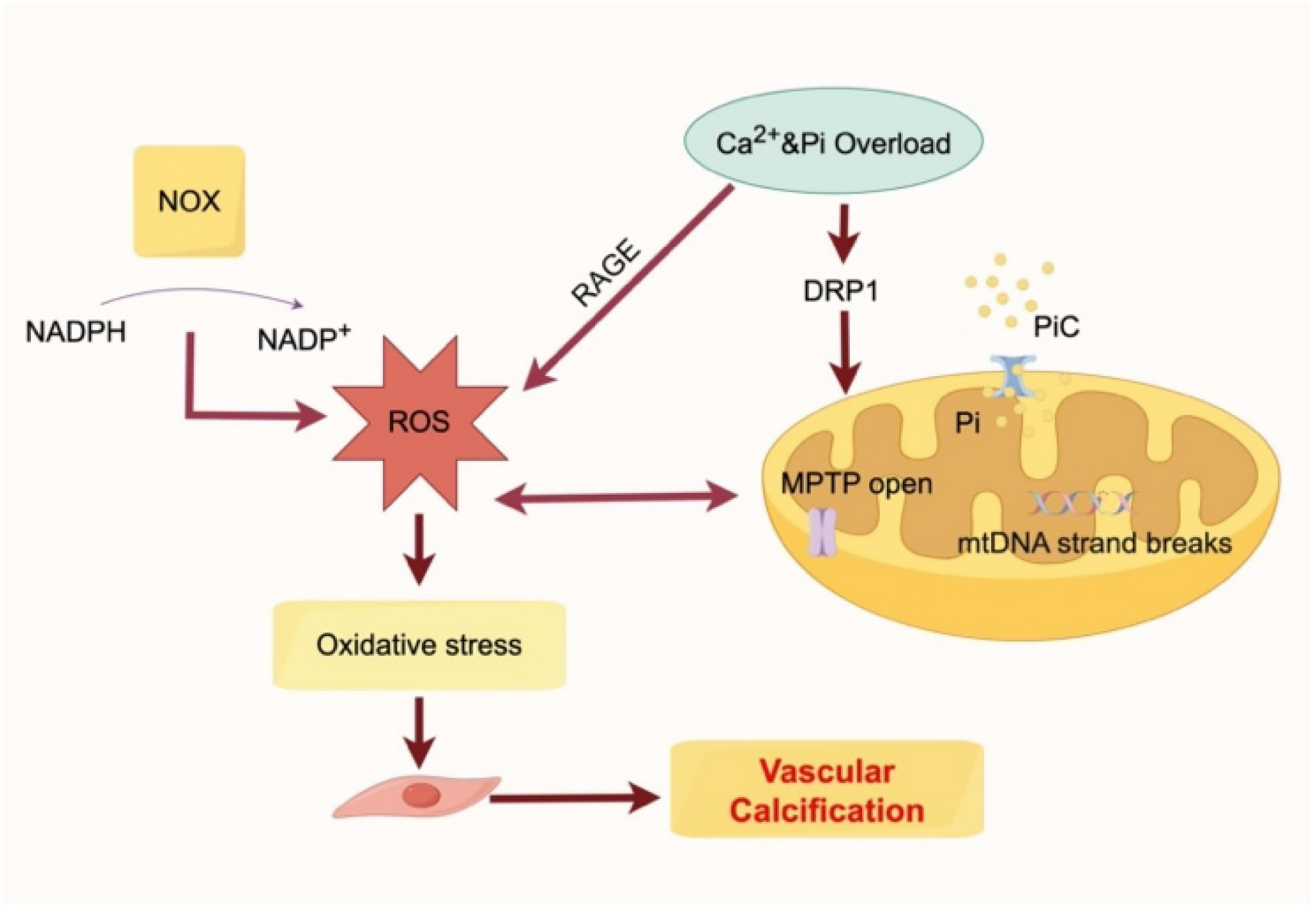
NOX drives excessive ROS production and contributes to oxidative stress. Ca^2+^ and Pi overload promote mitochondrial damage through DRP1-mediated pathways, opening of the MPTP, Pi transport via the PiC, and mtDNA strand breaks. The rapid production of RAGE ligands in response to Pi induces the activation of RAGE signalling. These events enhance oxidative stress and ultimately accelerate vascular calcification. NOX, NADPH oxidases; RAGE, receptor for advanced glycation end products; DRP1, dynamin-related protein 1; MPTP, mitochondrial permeability transition pore; mtDNA, mitochondrial DNA.

Phosphate transporters (PiT-1/-2) mediate Pi entry into VSMCs via sodium-dependent cotransport, while mitochondrial phosphate carriers (PiC) facilitate intramitochondrial Pi uptake (23). Excessive Pi uptake leads to mitochondrial hyperpolarization and superoxide overproduction (23). H_2_O_2_, a key ROS in atherosclerosis, induces VSMC osteogenic differentiation by upregulating RUNX2 (37). ROS accumulation further damages the mitochondrial outer membrane, causing Ca^2+^ overload and DNA injury (38). In addition, advanced glycation end products (AGEs) and their receptor (RAGE) significantly contribute to VC by amplifying oxidative stress (39). Pi-induced RAGE ligand production enhances oxidative stress, upregulates Pit-1 transcription, and increases RUNX2 expression (40). Meanwhile, NOX-derived ROS participate in AGE-mediated VSMC apoptosis, a critical mechanism in chronic kidney disease and diabetes-associated VC (41) (Figure 2).

Antioxidant systems are essential for maintaining vascular health. Dietary antioxidants, particularly polyphenols, can modulate the uncoupling of endothelial nitric oxide synthase (eNOS). In vascular diseases, eNOS uncoupling favors the generation of superoxide radicals rather than nitric oxide. Polyphenols mitigate oxidative stress and improve vascular endothelial dysfunction (VED) by scavenging free radicals or inhibiting radical-generating pathways (42).

## 3 Epigenetic regulation and oxidative stress in vascular calcification

Current research on oxidative stress and epigenetics in VC emphasizes their complex interaction. In aging, studies reveal that oxidative stress and epigenetic changes—including DNA methylation, histone modifications, and non-coding RNAs—play a role in the molecular mechanisms behind age-related decline (43). In cancer biology, more focus is being placed on how oxidative stress alters the epigenetic machinery, thereby encouraging tumor initiation, progression, and chemoresistance. Understanding these relationships may lead to new therapeutic strategies (44).

Epigenetics refers to heritable changes in gene expression without alterations in the DNA sequence, including DNA methylation, histone modifications, and regulation by non-coding RNAs (45, 46). Accumulating evidence links ROS with epigenetic modifications in VC.

### 3.1 DNA methylation

DNA methylation is regulated by DNA methyltransferases (DNMTs) and ten–eleven translocation (TET) family dioxygenases. Usually, CpG island methylation in gene promoters is linked to transcriptional silencing. Typically, CpG island methylation in gene promoters is associated with transcriptional silencing (47). TET proteins are Fe (II)/α-ketoglutarate (α-KG)-dependent dioxygenases that oxidize 5-methylcytosine (5mC) into 5-hydroxymethylcytosine (5hmC) and subsequent products (48). TET activity can be inhibited by 2-hydroxyglutarate (2-HG). α-KG, a tricarboxylic acid (TCA) cycle intermediate, is generated by isocitrate dehydrogenases (IDHs). Their activity is inhibited by 2-HG, which is produced by mutant IDHs through aberrant oxidation of isocitrate. This results in TET inhibition, DNA hypermethylation, increased ROS generation, and enhanced oxidative stress sensitivity (49, 50).

#### 3.1.1 ROS-mediated DNA methylation changes in VC

TET2 overexpression promotes VSMC differentiation by enhancing contractile gene expression and reducing DNA methylation (51). Recent studies also show that the α-KG mitigates VC by activating TET2, which in turn suppresses NLR family pyrin domain containing 3 (NLRP3) inflammasome signaling (52). High-phosphate conditions upregulate DNMTs, increase smooth muscle 22α (SM22α) promoter methylation, downregulate SM22α expression, and enhance RUNX2 expression and mineral deposition. These findings underscore epigenetic SM22α methylation as an early event in VC (53). Numerous studies confirm that oxidative stress activates and upregulates DNMTs (54, 55). Consistently, Li et al. demonstrated that H_2_O_2_ enhances osteogenic transdifferentiation of VSMCs by reducing ALP and RUNX2 methylation, an effect reversible by DNMT3a overexpression (56).

#### 3.1.2 DNA methylation-mediated ROS changes in VC

DNA methylation can also affect ROS homeostasis. Folate supplementation prevents atherosclerosis by lowering homocysteine levels, increasing the S-adenosylmethionine (SAM)/S-adenosylhomocysteine (SAH) ratio, and enhancing DNMT activity (29). Folate further protects against oxidative damage and apoptosis in ApoE−/− mice by promoting DNMT activity, increasing methylation of the vascular peroxidase 1 (VPO1) promoter, and reducing VPO1 expression, thus providing vascular protection (57). MtDNA methylation also plays a role in redox regulation. Studies suggest that DNMTs can methylate mtDNA when SAM enters mitochondria (58). Liu et al. demonstrated that platelet-derived growth factor (PDGF)-BB stimulation causes DNMT1 to translocate into mitochondria, where it methylates the mtDNA D-loop. This process suppresses mtDNA transcription, impairs mitochondrial function, reduces ATP production, and results in VSMC dedifferentiation and loss of the contractile phenotype (59). These findings strengthen the link between DNA methylation dynamics, ROS, and VC (60, 61).

### 3.2 Histone modifications

Histone modifications have significant effects on vascular cells. In eukaryotes, nucleosomes are made up of DNA wrapped around histone octamers, with histone “tails” extending outward that undergo diverse post-translational modifications. These include acetylation, methylation, phosphorylation, ubiquitination, and sumoylation, collectively known as histone modifications. Increasing evidence suggests that histone modifications are closely associated with vascular calcification (62).

#### 3.2.1 Histone acetylation

Histone acetylation is a dynamic and reversible process regulated by histone acetyltransferases (HATs), which add acetyl groups, and histone deacetylases (HDACs), which remove them. Acetyl-CoA acts as the donor of acetyl groups and functions both as a metabolic intermediate and as a signaling molecule in maintaining homeostasis (63). HATs and HDACs regulate the expression of genes involved in VSMC contractility, differentiation, extracellular matrix deposition, and responses to vasoactive stimuli such as angiotensin II (64).

##### 3.2.1.1 ROS-Mediated histone acetylation changes in VC

HDACs are essential in controlling the osteogenic transition of VSMCs (65, 66). For example, HDAC1 suppresses lysine-specific demethylase 1 (LSD1) transcription via H3K9ac modification at the LSD1 promoter, activating autophagy through the mechanistic target of rapamycin (mTOR) pathway and ultimately attenuating VC (67). Similarly, HDAC8 inhibits osteogenic differentiation by suppressing H3K9 acetylation and RUNX2 expression (68, 69). Accumulating evidence suggests that oxidative stress alters HDAC activity (70). Wu et al. reported that H_2_O_2_ can inhibit HDACs such as HDAC1 and HDAC6, thereby affecting downstream gene acetylation (71–73). Moreover, NOX4-mediated oxidative stress promotes oxidative modification and nuclear translocation of HDAC4, reducing its inhibitory effect on transcription (74). In VSMCs, cytosolic HDAC4 interacts with cytoskeletal proteins such as ENIGMA (Pdlim7), a process essential for VC development (65). Thus, oxidative stress may promote VC progression by controlling HDAC4 localization and activity.

Acetyl-CoA, derived from glucose, fatty acids, or acetate, not only fuels ATP production but also serves as the primary substrate for histone acetylation. It serves as the primary substrate for histone acetylation. The majority of cytosolic acetyl-CoA is supplied by mitochondrial metabolism through ATP citrate lyase (ACLY), while acetyl-CoA synthetase 2 (ACSS2) provides an additional source from acetate (63). Consequently, the availability of acetyl-CoA establishes a link between cellular energy metabolism and epigenetic regulation, presenting a vital mechanism through which metabolic states can influence vascular calcification. Shao et al. show that the inhibition of acyl-CoA synthetase blocks the mineralization of VSMC (75).

##### 3.2.1.2 Histone acetylation-mediated ROS changes in VC

Conversely, histone acetylation can control ROS production. Sirtuins (SIRTs), a class of NAD+-dependent lysine deacetylases, serve as important redox signaling molecules. Mitochondria play an important role in regulating the cellular NAD^+^/NADH ratio, which in turn controls the activities of sirtuins. By deacetylating transcription factors, SIRTs regulate the expression of enzymes that generate ROS and antioxidant defenses (76, 77). Multiple studies have identified SIRTs as key effectors in oxidative stress signaling (78–80). Overexpression of SIRT1 protects against H_2_O_2_-induced vascular dysfunction and premature aging by deacetylating p53, which results in decreased plasminogen activator inhibitor-1 (PAI-1) expression and increased eNOS activity (81, 82). SIRT1 activation has also been shown to reduce NOX-derived ROS, thus providing antioxidant and anti-aging benefits in the cardiovascular system (83, 84). Notably, SIRT1 reverses H_2_O_2_-induced DNA damage and calcification, highlighting its role in counteracting oxidative stress (1). Luteolin, a natural tetrahydroxyl flavonoid, can protect against vascular calcification by modulating the Sirtuin1 (SIRT1)/CXC Chemokine Receptor 4 (CXCR4) signaling pathway and promoting autophagy. In rats, luteolin significantly improved vascular calcification induced by a high-fat diet and vitamin D3. *In vitro*, it repressed the formation of mineralized nodules and ALP activity in H_2_O_2_-treated VSMCs (85). Thus, luteolin may inhibit oxidative stress-induced vascular calcification by activating SIRT1-mediated regulation. HDACs also interact with oxidative stress during VSMC osteogenic differentiation. For example, Bai et al. reported that HDAC5 inhibition reduced angiotensin II–induced oxidative stress in VSMCs (86).

Collectively, these findings indicate that histone acetylation not only mediates ROS-induced transcriptional changes but also provides feedback to control oxidative stress, thereby supporting the epigenetic–redox interaction in VC.

#### 3.2.2 Histone methylation

Histone methylation, a major epigenetic modification, is controlled by histone methyltransferases (HMTs) and reversed by histone demethylases (HDMs). Lysine methylation is the main type in eukaryotes, with common methylation sites including H3K4, H3K9, H3K27, H3K36, H3K79, and H4K20 (87). Two major HDM families regulate histone demethylation: the LSD family and the Jumonji C (JmjC) domain-containing family (JMJD) (88). For example, Kang et al. reported that JMJD2B/KDM4B promotes osteogenic differentiation of VSMCs by lowering H3K9me2 levels at the RUNX2 promoter (89). Similar to DNA methylation, histone methylation requires SAM as the methyl donor, linking mitochondrial function to histone methylation through SAM synthesis. JMJD enzymes depend on Fe (II), oxygen, and α-KG, and are inhibited by fumarate and succinate. Mitochondrial dysfunction can cause excessive histone methylation, partly due to increased ROS, which impairs HDM activity (90). Cumulative evidence shows that histone methylation is closely linked to the initiation and progression of VC, affecting processes such as metabolic reprogramming, apoptosis, oxidative stress, and multiple signaling pathways (91).

##### 3.2.2.1 ROS-Mediated histone methylation changes in VC

SET domain–containing 7 (SETD7), a histone methyltransferase, promotes nuclear factor kappa-B (NF-κB) activation and pro-inflammatory cytokine production via H3K4me1-dependent transcription in response to ROS (92). NF-κB signaling, in turn, plays a central role in phosphate-induced VC (93–95). Intracellular ROS can activate NF-κB, which regulates genes involved in atherosclerosis and inflammation, including interleukin-6 (IL-6) (96). Kurozumi et al. showed that IL-6 recruits JMJD2B to the RUNX2 promoter, reducing H3K9me3 and promoting VSMC calcification (97). Moreover, adenosine-mediated activation of AMP-activated protein kinase (AMPK), a central regulator of cellular energy balance (98), inhibits DNMT3b and leads to hypomethylation of the H19 promoter and decreases RUNX2 expression, thereby mitigating VSMC osteogenic differentiation (99). Since AMPK is a vital energy sensor in cellular metabolism, especially during metabolic stresses like oxidative stress, these findings imply a mechanistic connection between oxidative stress, histone methylation, and VC (100).

##### 3.2.2.2 Histone methylation-mediated ROS changes in VC

**Figure 3:**
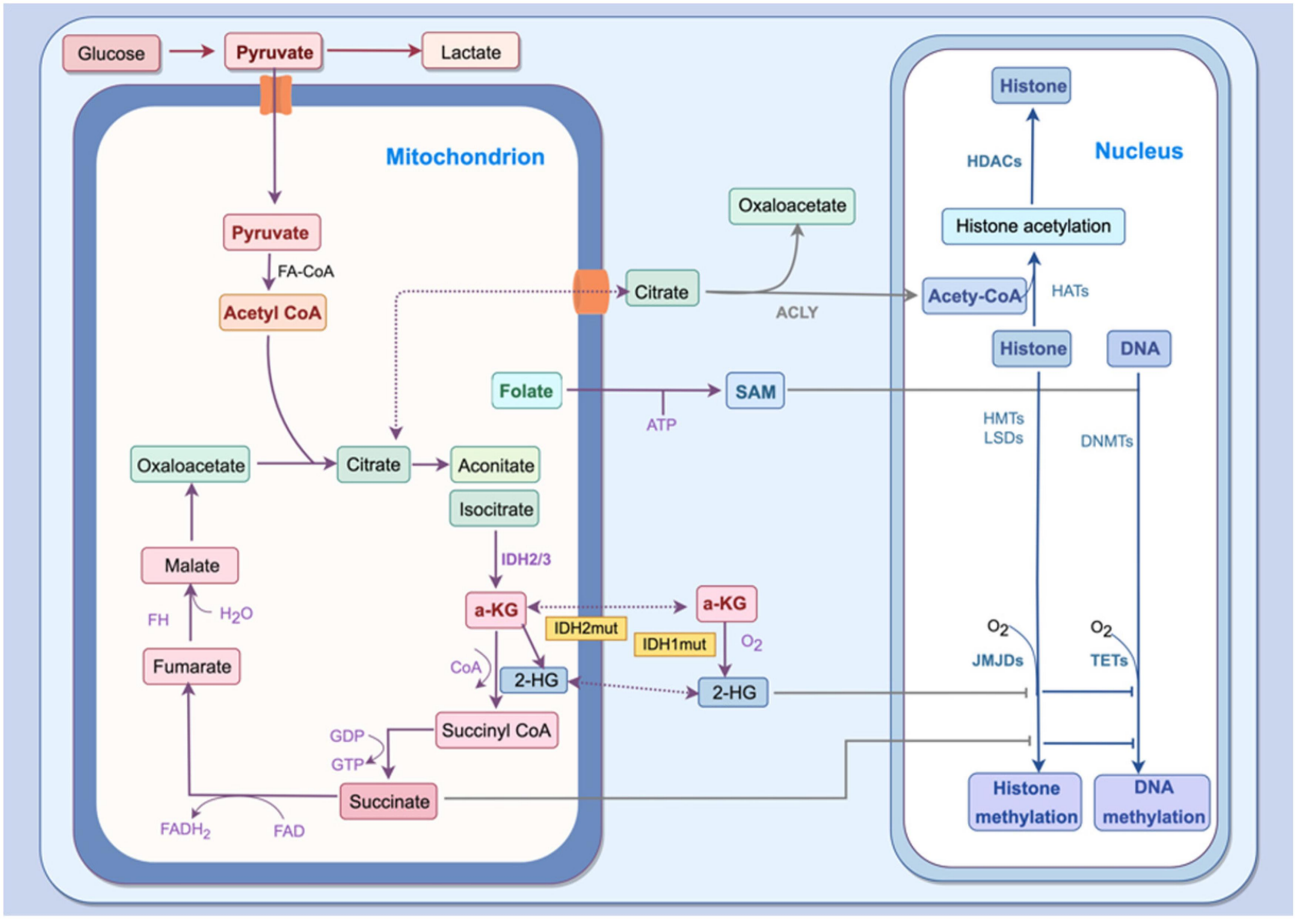
Mitochondrial metabolism tightly links oxidative stress to epigenetic regulation. Pyruvate-derived acetyl-CoA (supplied to the cytosol by ACLY) promotes histone acetylation, whereas SAM produced by folate metabolism serves as the methyl donor for DNA and histone methylation. α-KG is a co-substrate of TET and JMJD demethylases, which is generated by IDHs. It follows that metabolic changes driven by oxidative stress shape the epigenetic landscape. ACLY, ATP citrate lyase; α-KG, alpha-Ketoglutarate; 2-HG, 2-hydroxyglutarate; IDH, isocitrate dehydrogenase; FH, fumarate hydratase; SAM, S-adenosylmethionine; JMJD, Jumonji C domain-containing; TET, ten-eleven translocation; DNMT, DNA methyltransferase; HMT, histone methyltransferase; LSD, Lys-specific demethylase; HDAC, histone deacetylase.

Histone methylation can also regulate oxidative stress. Hypoxia-inducible factor-1α (HIF-1α) stabilization depends on mitochondrial ROS (101), and its activation promotes RUNX2 expression and VC (101, 102). N-acetylcysteine, a ROS scavenger, inhibits extracellular matrix calcification by suppressing HIF-1α expression (103). SETD7 has been identified as a negative regulator of HIF-1α transcriptional activity (104, 105), and Liu et al. demonstrated that SETD7 inhibits HIF-1α-mediated genes involved in metabolic reprogramming. The knockdown of SETD7 increases glucose uptake and intracellular ATP levels (104). Furthermore, SETD7 regulates ROS signaling by inhibiting peroxisome proliferator-activated receptor-γ coactivator 1α (PGC1α) and antioxidant enzymes such as SOD2 and catalase (92). Together, these findings suggest that histone methylation not only responds to oxidative stress but also actively regulates ROS production, thereby contributing to VC (Figure 3).

### 3.3 MicroRNAs (miRNAs)

MicroRNAs (miRNAs) are small noncoding RNAs, about 20–24 nucleotides long, that suppress target gene expression by binding to the 3′ untranslated regions (UTRs) of messenger RNAs (mRNAs). Depending on their genomic location, miRNA genes are categorized as intronic, exonic, or intergenic (106). As post-transcriptional regulators, miRNAs are crucial for mRNA degradation and repression of translation (107). They are increasingly recognized as biomarkers and regulators in cardiovascular diseases, including VC.

**Figure 4:**
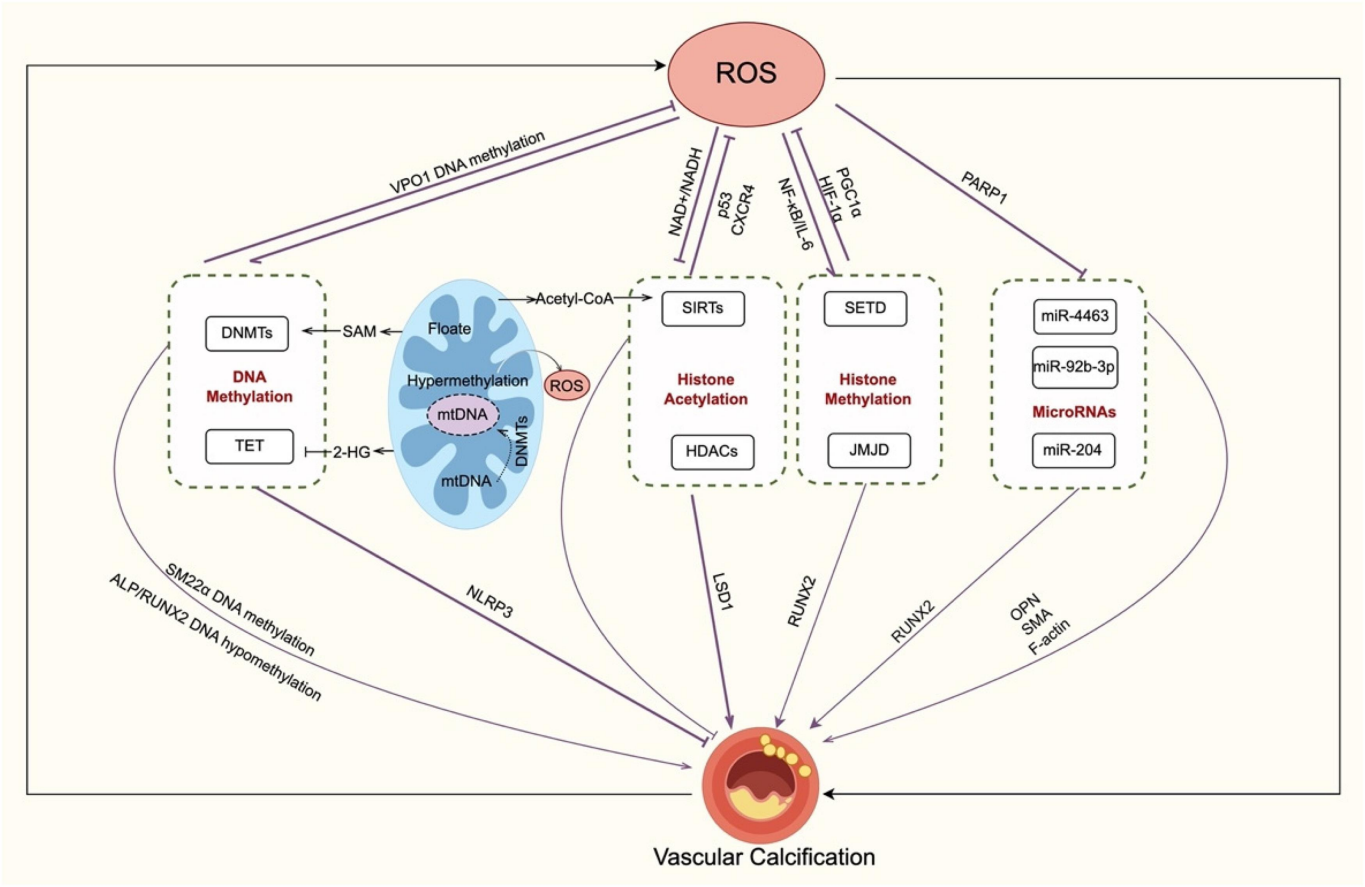
Crosstalk between oxidative stress and epigenetic regulation in VC. Oxidative stress and epigenetic mechanisms create a regulatory loop in VC. DNA methylation, ROS enhance DNMT activity, leading to the methylation of VSMC contractile genes (e.g., SM22α) and osteogenic activation (RUNX2, ALP). Additionally, DNA methylation influences ROS through folate–SAM pathways, VPO1 suppression, and mitochondrial DNA methylation. Histone modifications, ROS change HDAC localization and activity and suppress LSD1 transcription through histone acetylation, reducing vascular calcification; sirtuins (e.g., SIRT1) counteract oxidative stress and inhibit VC. Histone methylation (e.g., SETD, JMJD2B) connects ROS signals with NF-κB, HIF-1α, PGC-1α, and RUNX2 regulation, linking inflammation, energy metabolism, and calcification. MicroRNAs, PARP1 suppresses miR-204 during oxidative stress. ROS-sensitive miRNAs (e.g., miR-4463, miR-92b-3p, miR-204) regulating VSMC osteogenic transformation. transformation. Overall, oxidative stress not only induces but is also modulated by DNA methylation, histone modifications, and non-coding RNAs, driving VC progression. VC, vascular calcification; ROS, reactive oxygen species; DNMT, DNA methyltransferase; TET, ten–eleven translocation protein; SAM, S-adenosylmethionine; 2-HG, 2-hydroxyglutarate; VSMC, vascular smooth muscle cell; ALP, alkaline phosphatase; RUNX2, runt-related transcription factor 2; NLRP3, NLR family pyrin domain containing 3; VPO1, vascular peroxidase 1; mtDNA, mitochondrial DNA; HAT, histone acetyltransferase; HDAC, histone deacetylase; JMJD, Jumonji C domain-containing; SETD, SET domain–containing; CXCR4, CXC chemokine receptor 4; SIRT, sirtuin; AMPK, AMP-activated protein kinase; NF-κB, nuclear factor kappa-B; IL-6, interleukin-6; PGC1α, peroxisome proliferator-activated receptor-γ coactivator 1α; HIF-1α, hypoxia-inducible factor-1α; LSD, lys-specific demethylase; miRNA, microRNA; PARP1, Poly (ADP-ribose) polymerase 1; OPN, osteopontin; SMA, smooth muscle actin;.

Oxidative stress significantly impacts miRNA expression, affecting VSMC function and phenotype. For instance, miR-4463 regulates VSMC phenotypic switching under oxidative stress. When miR-4463 is downregulated, it increases osteopontin (OPN) expression while decreasing smooth muscle actin (SMA) and F-actin, thereby promoting calcification (108). Basic fibroblast growth factor (bFGF), a potential miR-4463 target, promotes VSMC migration through ROS production (109). Similarly, downregulation of miR-92b-3p reduces hypoxia-induced VSMC proliferation by inhibiting the mTOR pathway (110). Poly (ADP-ribose) polymerase 1 (PARP1) also interacts with miRNAs in VC. PARP1 suppresses miR-204 expression, thereby enhancing RUNX2 expression and promoting VSMC osteogenic transformation (111). Excessive PARP1 activation during oxidative stress leads to mitochondrial membrane depolarization (112). Therefore, the PARP1–miR-204–RUNX2 axis is a crucial connection between oxidative stress, miRNA regulation, and VC. More generally, oxidative stress influences the expression of many miRNAs, which then regulate redox sensors and adjust antioxidant defenses (113) (Figure 4).

## 4 Summary

Vascular calcification is a hallmark of advanced cardiovascular disease, caused by VSMC phenotypic switching from a contractile to an osteogenic state marked by RUNX2, MSX2, and ALP expression (8, 9). Oxidative stress and epigenetic reprogramming serve as central mechanisms in this process. Phosphate overload increases TCA cycle activity, leading to higher mitochondrial ROS production and connecting metabolic intermediates like acetyl-CoA and SAM to epigenetic regulation (114, 115). DNA methylation, influenced by ROS-regulated DNMT and TET activity, modifies key genes such as SM22α and RUNX2, while mtDNA methylation exacerbates mitochondrial dysfunction (51, 53–61). Histone acetylation/deacetylation (*via* HDACs and SIRTs) and histone methylation (e.g., H3K9, H3K4) regulate RUNX2, HIF-1α, and NF-κB pathways (67–73, 89–92, 94–98, 116–119). Additionally, miRNAs modulate the VSMC phenotype and oxidative stress responses, thereby reinforcing the feedback loop between ROS and epigenetic changes.

Considering the essential physiological roles of epigenetic mechanisms, non-specific inhibitors present therapeutic challenges. Future research should clarify how ROS, chromatin modifications (such as H3K9me3 and H3K4me1), and non-coding RNAs interact in VC, with focus on metabolic intermediates that connect energy status to epigenetic programming. Targeting this redox–epigenetic axis could lead to new strategies for preventing VC and associated cardiovascular diseases.
